# Development of High-Sensitivity Piezoresistive Sensors Based on Highly Breathable Spacer Fabric with TPU/PPy/PDA Coating

**DOI:** 10.3390/polym14050859

**Published:** 2022-02-22

**Authors:** Xiujuan Wang, Xiaoyu Gao, Yu Wang, Xin Niu, Tanyu Wang, Yuanjun Liu, Fangxi Qi, Yaming Jiang, Hao Liu

**Affiliations:** 1 School of Textile Science and Engineering, Tiangong University, Tianjin 300387, China; xjzwang@outlook.com (X.W.); gxy0822@yeah.net (X.G.); wyu0209@163.com (Y.W.); newhsin@163.com (X.N.); Wangtanyu2019@163.com (T.W.); liuyuanjunsd@163.com (Y.L.); qfx2020786971@163.com (F.Q.); 2 Aerospace Life-supports Industries LTD, Xiangyang 441003, China; 3 Aviation Key Laboratory of Science and Technology on Life-support Technology, Xiangyang 441003, China; 4 Institute of Smart Wearable Electronic Textiles, Tiangong University, Tianjin 300387, China; 5 Key Laboratory of Advanced Textile Composite Materials of Ministry of Education, Tiangong University, Tianjin 300387, China

**Keywords:** space fabric, Polypyrrole (PPy), piezoresistive sensors, thermoplastic polyurethane (TPU) coating

## Abstract

In recent years, the research of flexible sensors has become a hot topic in the field of wearable technology, attracting the attention of many researchers. However, it is still a difficult challenge to prepare low-cost and high-performance flexible sensors by a simple process. Three-dimensional spacer fabric (SF) are the ideal substrate for flexible pressure sensors due to its good compression resilience and high permeability (5747.7 mm/s, approximately 10 times that of cotton). In this paper, Thermoplastic polyurethane/Polypyrrole/Polydopamine/Space Fabric (TPU/PPy/PDA/SF) composite fabrics were prepared in a simple in-situ polymerization method by sequentially coating polydopamine (PDA) and Polypyrrole (PPy) on the surface of SF, followed by spin-coating of different polymers (thermoplastic polyurethane (TPU), polydimethylsiloxane (PDMS) and Ecoflex) on the PPy/PDA/SF surface. The results showed that the TPU/PPy/PDA/SF pressure sensors prepared by spin-coating TPU at 900 rpm at a concentration of 0.3 mol of pyrrole monomer (py) and a polymerization time of 60 min have optimum sensing performance, a wide working range (0–10 kPa), high sensitivity (97.28 kPa^−1^), fast response (60 ms), good cycling stability (>500 cycles), and real-time motion monitoring of different parts of the body (e.g., arms and knees). The TPU/PPy/PDA/SF piezoresistive sensor with high sensitivity on a highly permeable spacer fabric base developed in this paper has promising applications in the field of health monitoring.

## 1. Introduction

In recent years, flexible wearable pressure sensors have attracted widespread research interest due to their great potential for human activity detection, human-computer interaction interfaces, medical health testing, and artificial electronic skin [1,2]. By attaching flexible strain pressure sensors to the joints, wrists, and heart of the human, real-time monitoring of human movement status [3], pulse/heart rate [4], and ECG and EEG signals [5,6] and heart valves [2] can be achieved, providing early warning of the onsets of diseases. So far, piezoresistive [7,8], capacitive [9], and self-powered [10,11] sensors have been reported. Among these, piezoresistive sensors, which convert external pressure or strain into resistance change signals, have become ideal for high-performance flexible wearable sensors because of their easy signal acquisition, low manufacturing cost, simple assembly process, and excellent sensing performance [7]. Piezoresistive sensors are mainly constructed using thin-film and three-dimensional structures. Among those, thin-film piezoresistive sensors are usually made by coating elastic media such as polydimethylsiloxane (PDMS) [12,13], thermoplastic polyurethane (TPU) [14,15], Ecoflex [16,17], polymethyl methacrylate (PMMA) [18,19], polyethylene terephthalate (PET) [2], and cyclic olefin copolymer (COC) [20] as the substrate material. Montazerian et al. [12], Ghahramani et al. [13], Kumar et al. [14], and He et al. [17] used flexible polymers such as PDMS and TPU as substrates and coated them with conductive chopped carbon fiber (CCF), multi-walled carbon nanotubes (MWCNTs) and polypyrrole (PPy). Flexible pressure sensors were prepared, and the measurement accuracy of the sensors could reach about 20 kPa^−1^ for medical health monitoring and wearable electronics. Of interest, Fernandez et al. [2] prepared Fe/Ni high-sensitivity pressure sensors based on the giant magneto-impedance (GMI) effect, using COC as a substrate and magnetron sputtering deposition method, with sensitivity up to 1 Ω/Pa at 0–1 Pa; Melzer et al. [20] constructed high-sensitivity giant magneto resistive (GMR) sensor elements on ultrathin 1.4 mm PET foil, which can be uniaxially or biaxially stretched on elastic support with strain up to 270% and withstand more than 1000 cycles without fatigue, and can be used in soft robotics, medical health monitoring, and electronic skin fields. Lu et al. [21] developed a highly sensitive interlocking piezoresistive sensor based on an ultrathin ordered nanocone array film using the template method, which exhibited a pressure range of 0–200 Pa range exhibited a high sensitivity of 268.36 kPa^−1^, an ultra-low detection limit of 0.98 Pa, a fast response time of 48 ms, and a recovery time of 56 ms.

The 3D-structured piezoresistive sensors mostly use lightweight, soft, and highly elastic 3D porous sponge as the substrate to prepare highly sensitive sensors [8,22,23]. Wu et al. [22] successfully prepared an elastic 3D conductive network using gold ion sputtering and cleverly designed channel cracks in the 3D sponge skeleton to achieve fast response time (9 ms), ultra-low detection limit (0.568 Pa), and good reproducibility over 1000 cycles, making it promising for biological motion and health monitoring applications. Spacer fabrics with three-dimensional are also being used in an increasing number of applications because of their excellent properties, such as sensing substrate materials [24], self-generating substrates [11,25], and protective cushioning layers for the human body [26,27]. Li et al. [24] coated carbon black uniformly on a slightly cured silicone elastomer pre-adhered to the surface of a 3D fabric and fabricated a pressure sensor with good conductivity that can measure external pressure up to 283 kPa with a conductivity range of 9.8 kΩ and a sensitivity of 50.31 × 10^−3^ kPa^−1^, which is superior to other fabric-based pressure sensors. Wu et al. [25] successfully prepared an all-fabric pressure sensor with a wireless battery-free monitoring system using a 3D spacer fabric, in which a highly conductive fabric electrode sandwiched between two three-dimensional penetration fabrics was used as the dielectric layer. Due to the good elastic recovery of the spacer fabric, the capacitive pressure sensor exhibited a sensitivity of 0.283 KPa^−1^, fast response time and good cycling stability (>=20,000). Rajan et al. [26] used the plasma treated spacer fabric as insoles and investigated their thermal comfort properties, showing that the spacer insole had good thermal physiological comfort as insoles. Kumar et al. [27] used spacer fabric instead of foam in a motorcycle helmet liner and studied the breathability, heat, and water vapor resistance and compression properties of the spacer fabric, which showed that spacer fabric has better energy absorption, breathability, and evaporative heat transfer. Based on the high permeability, good compression resilience [28,29], and good thermal and moisture physiological comfort [27] of spacer fabrics, they have become ideal for use as a flexible sensor substrate in recent years.

Spacer fabrics (SF) are usually knitted with non-conductive polymers such as polyester and therefore need to be coated with conductive materials to build a three-dimensional conductive network. Commonly used conductive materials include metal particles, carbon-based materials, and conductive polymers, of which, conductive polymers are good candidates for piezoresistive pressure sensors due to their excellent conductivity and good biocompatibility. Commonly used conductive polymers include polypyrrole (PPy) [30,31,32], polythiophene [33], polyaniline [34], etc. Among these, polypyrrole (PPy) is a good candidate for the preparation of sensors due to its simple synthesis process, low cost, high oxidation resistance, high conductivity and specific capacitance values, and easy film formation [35,36]. Through in situ polymerization, PPy can form continuous coatings or thin films on a variety of materials. Due to the inherent stiffness and brittleness of the thin film coating consisting of dense PPy particles, they tend to peel off during cyclic compressive use, resulting in degradation of the sensor’s performance. Therefore, the manufacture of flexible PPy pressure sensors with high sensitivity and stable performance remains challenging.

In this paper, a multi-coating preparation of pressure sensors based on three-dimensional spacer fabrics is proposed. Firstly, polypyrrole conductive spacer fabrics with excellent electrical conductivity were obtained by in situ coating with polypyrrole after self-polymerization modification of the spacer fabric fiber surface with dopamine, denoted as PPy/PDA/SF. To further improve the durability and mechanical stability of the conductive fabrics, we propose a process for impregnating and spin-coating polymers on the surface of PPy/PDA/SF, and investigate in detail the optimal impregnation and spin-coating process for different polymers. Thanks to the unique structure of the 3D spacer fabric with high surface area and abundant contact points, it can form a homogeneous 3D porous conductive network in the composite fabric and thus has excellent sensitivity to fine pressure. As a result, the sensors prepared in this study have low detection limits, high sensitivity, fast response, good cycling stability and reliability, moreover maintain good compression resilience and permeability of their substrate, thus offering promising applications in the field of health monitoring.

## 2. Materials and Methods

### 2.1. Materials

SF was purchased from Changshu Zhengfangyi Knitting Co., Ltd., Changshu, China., with a density of 0.3 ± 0.02 g/cm^2^. Dopamine hydrochloride (DA, 98%) was purchased from Shanghai Aladdin Biochemical Technology Co., Ltd., Shanghai, China. Pyrrole was purchased from Shanghai Meryer, Shanghai, China. Anhydrous ethanol, tris (hydroxymethyl) aminomethane (Tris basis, 99%), dimethylformamide (*N*,*N*-dimethylformamide DMF) were purchased from Tianjin Kemiou Chemical Reagent Co., Ltd., Tianjin, China. Ferric chloride was purchased from Tianjin Fengchuan Chemical Reagent Technology Co., Ltd., Tianjin, China. P-toluenesulfonic acid was purchased from Tianjin Guangfu Technology Development Co., Ltd., Tianjin, China, TPU particles (type 60A), Ecoflex, were purchased from BASF, Germany, and polydimethylsiloxane (PDMS) was purchased from Dow Corning, USA. All chemicals were used without further purification.

### 2.2. Fabrication of PPY/PDA/SF and TPU/PPY/PDA/SF

Preparation of PDA/SF composite fabric

SF was cut to 2 cm × 2 cm and the fabric was ultrasonically cleaned with acetone and deionized water in turn to remove impurities and grease. A dopamine solution with a pH of 8.5 was prepared by dissolving 0.4325 g of tris(hydroxymethyl) aminomethane (Tris base) and 2 g of dopamine hydrochloride (DA) in 500 mL of ethanol solution (V_Ethanol_:V_H2O_ = 7:3). The washed SF was immersed in the dopamine solution (bath ratio 1:50) at room temperature and stirred for 24 h, during which time the DA self-polymerized on the fabric surface to form polydopamine (PDA), then the treated fabric was removed, washed in deionized water, and dried at room temperature to obtain PDA/SF.

Preparation of PPY/PDA/SF composite fabric

A certain amount of pyrrole (Py) monomer and distilled water were mixed to prepare different concentrations of pyrrole solution (0.1 mol/L, 0.2 mol/L, 0.3 mol/L, and 0.4 mol/L, respectively), and stirred at constant speed on a magnetic stirrer until the pyrrole was evenly mixed with water, and then the dopamine-treated spacer fabric (PDA/SF) was immersed in an aqueous solution of pyrrole monomer and stirred at constant speed for 30 min at room temperature (RT). The corresponding concentrations of ferric chloride and p-toluenesulfonic acid (molar ratio of ferric chloride to pyrrole is 3:2) were then added to 30 mL of deionized water and stirred until the drugs were completely dissolved to obtain the oxidizing and doping agents for the reaction. The configured oxidizing and doping agents were slowly dripped into the pyrrole monomer solution to initiate polymerization to form PPy coating on the PDA/SF. The polymerization reaction was carried out on a magnetic stirrer at room temperature for 1 h. The interval fabric was washed with water and dried at room temperature, recorded as PPY/PDA/SF.

Preparation of TPU/PPY/PDA/SF composite fabric

TPU particles and *N*,*N*-dimethylformamide DMF solution were mixed in a conical flask according to the set mass fraction and then sealed, and the transparent and clarified TPU solution was obtained by stirring at 60 °C for 4 h. PDMS was prepared in the ratio of 10:1 for polymer, and Ecoflex was prepared in the ratio of 1:1 for Ecoflex polymer.

PPY/PDA/SF composite fabric was immersed in the polymer (TPU, PDMS, and Ecoflex) solution for 5 min, removed and placed in a centrifuge for 3 min at different speeds (600 rpm, 900 rpm, and 1200 rpm respectively), then dried in a vacuum-drying oven at 80 °C for 5 min. After repeating the above centrifugation/drying steps twice. The composite fabrics were dried in a vacuum-drying oven at 80 °C for 2 h. The resulting specimens were recorded as TPU/PPy/PDA/SF, PDMS/PPy/PDA/SF, and Ecoflex/PPy/PDA/SF, respectively.

**Figure 1:**
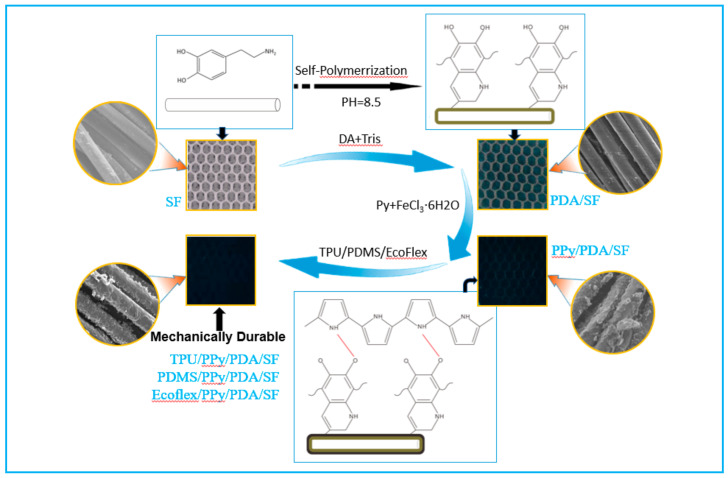
Schematic for the fabrication of the TPU/PPy/PDA/SF fabric.

Schematic illustration of the fabrication process of TPU/PPy/PDA/SF composite fabric was shown in Figure 1.

### 2.3. Characterization

A Regulus 8100 cold field emission scanning electron microscope from Hitachi was used to observe the uniformity of the pyrrole coating of the composite fabric and the surface morphology of the TPU after dip coating. The electrical conductivity of the composite fabric was measured using a U3402A multimeter from Agilent Technologies. The composite fabric was placed between two copper sheets with a 20 g weight placed on the copper sheet, and the two copper sheets were welded with wires leading to electrode lines connected to an Agilent multimeter to collect the resistance of the composite fabric. The infrared spectra of SF, PDA/SF, PPY/PDA/SF, and TPU/PPY/PDA/SF fabrics were measured using a Thermo Fisher Scientific Nicolet iS10 Fourier infrared spectrometer. The air permeability of PPY/PDA/SF composite fabrics and cotton fabrics were measured using a fully automatic air permeability meter (YG461H, Ningbo Textile Instruments Co., Ltd., Ningbo, China). Fabric mechanical properties and sensing properties were tested by using the TP-550 flexible sensor tester developed by Tianjin Zhirou Technology Co., Ltd., Tianjin, China to test the relationship between displacement and load and resistance, setting the compression displacement to 10%, 20%, 30%, 40%, and 50% of the fabric thickness and the compression speed to 0.1 mm/s, collecting the resistance of the fabric and the load change of the balance to reflect the softness and sensing properties of the fabric. The compression displacement is the same, the greater the compression the stiffer the fabric and vice versa. The fabric washing stability test was designed refer to the procedures of GB/T 8629-2017 for the washing experiment. A beaker was used to simulate the washing environment of the washing machine. A magnetic mixer was used as the driving force for rotation. The rotational speed of the magnetic mixer was set at 500 rpm and the four fabrics PPy/PDA/SF, TPU600/PPy/PDA/SF, TPU900/PPy/PDA/SF and TPU1200/PPy/PDA/SF were placed in the beaker for water washing and removed every 30 min, dried at 60 °C in a constant temperature blast dryer, weighed and resistance tested, and the resistance and weight after each water washing were recorded.

## 3. Results and Discussion

### 3.1. Mechanical Properties of SF

Two spacer fabrics commonly used in the market (SF-X and SF-L) were selected for comparative study of their mechanical properties to select a suitable spacer fabric as a substrate for pressure sensors.

**Figure 2:**
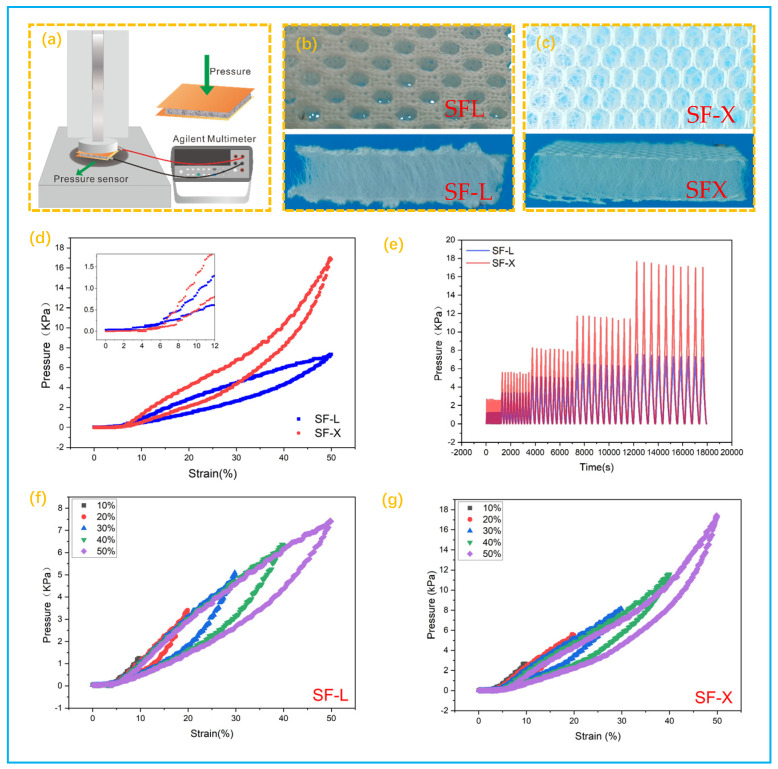
(**a**) Mechanical properties testing apparatus; (**b**) SF-L fabric; (**c**) SF-X fabric; (**d**) stress-strain curves of different spacer fabrics; (**e**) cyclic stability of different fabrics at different pressures; (**f**) hysteresis curves of SF-L fabric at different compression rates, (**g**) hysteresis curves of SF-X spacer fabric at different compression rates.

Figure 2a shows the schematic diagram of the measuring instrument. Figure 2b,c are the optical pictures of SF-L fabric and SF-X fabric respectively. It can be seen that the two fabric spacers have different filaments arrangements, but both show a typical porous honeycomb network structure as a whole. The elastic spacer filaments connect and support the upper and lower surfaces, enabling them to be used for a variety of pressure measurements and for the manufacture of comfortable motion monitoring sensors. The relationship between the forces on the scales of the two fabric substrates at the same compression is compared as shown in Figure 2d. When the strain is 50%, the stress of the SF-X fabric reaches 17 kPa which is significantly greater than the 7 kPa on the SF-L fabric, thus it can be concluded that SF-X fabric as the substrate can provide a relatively large range for the piezoresistive sensor. Figure 2e shows the cyclic stability test of fabrics with different compression amounts. The results show that the SF-X fabric has greater stress in the entire strain range, which further proves that the SF-X fabric can provide a wider working range for the pressure sensor. Figure 2f,g and Table S1 in the Supplementary Materials showed the comparison of hysteresis between SF-L fabric and SF-X fabric in different compressions, and the results show that SF-X fabric demonstrates lower hysteresis and zero drift. Therefore, combining the above mechanical property test results of the fabrics, SF-X fabric is more suitable as a substrate material for pressure sensors.

### 3.2. Coating Characterization

**Figure 3:**
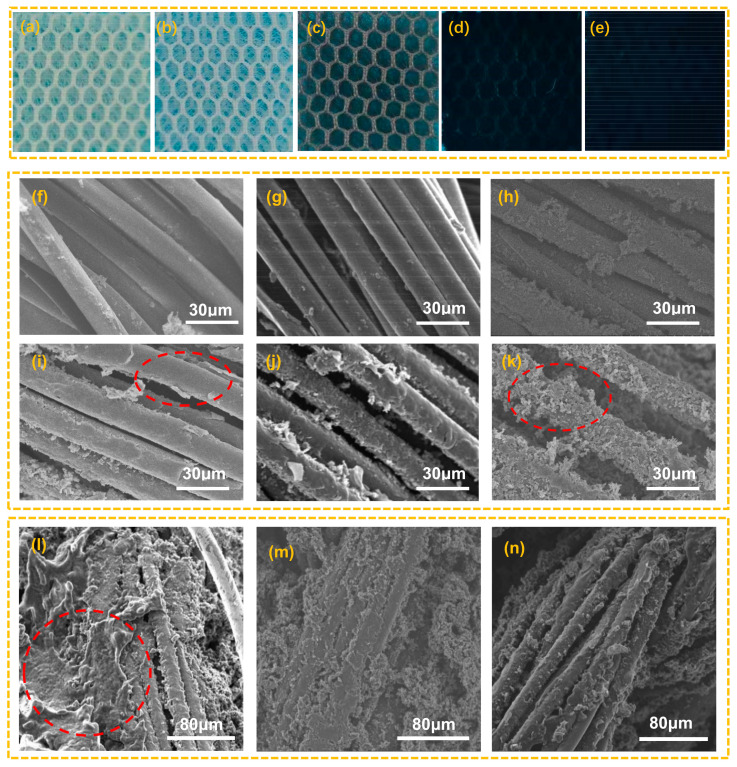
(**a**–**e**) are the optical images of original spacer fabric, cleaned spacer fabric, PDA/SF, PPy/PDA/SF and TPU/PPy/PDA/SF, (**f**) SEM of original spacer fabric, (**g**) SEM of PDA/SF, (**h**–**k**) PPy/PDA/SF with Py concentrations of 0.1–0.4 mol, (**l**) SEM of TPU600/PPy/PDA/SF, (**m**) SEM of TPU900/PPy/PDA/SF, (**n**) SEM of TPU1200/PPy/PDA/SF.

Figure 3 a–e shows the optical images of the original spacer fabric, the cleaned spacer fabric, the dopamine coating composite fabric PDA/SF, the pyrrole coating composite fabric PPy/PDA/SF, and the TPU coating composite fabric TPU/PPy/PDA/SF, respectively. As can be seen from the figure, the color of spacer fabric changed from white to light brown after dopamine plating and further changed to black after pyrrole plating. As can be seen by the SEM images of electron microscopy in Figure 3f–k, the fiber surface of the original spacer fabric is smooth, and the fiber surface shows a thin coating after dopamine plating, while the coating on the fabric surface after pyrrole plating has been clearly visible. Figure 3h shows the polypyrrole coated fabric prepared in pyrrole solution with the concentration of 0.1 mol/L. A small amount of polypyrrole was attached to the yarn as shown in the figure. When the concentration was increased to 0.2 mo1/L, dendritic polypyrrole grew on the surface of the yarn, while the coating is not yet complete (Figure 3i). When the concentration of pyrrole increased to 0.3 mo1/L, the surface of the fabric was more evenly and completely covered by the polypyrrole coating (Figure 3j). At a pyrrole concentration of 0.4 mo1/L, the polypyrrole coating appeared as a large agglomeration on the fabric surface and exhibited an irregular fork-like structure, resulting in an uneven polypyrrole coating on the fabric (Figure 3k). Figure 3l–n show the SEM images of TPU/PPy/PDA/SF with 4% wt. TPU concentration at different rotational speeds. On the microscopic level, TPU successfully bonded with PPy/PDA/SF, with adhesion becoming more evident at lower rotational speeds, while at higher speeds, no adhesion was evident. On a macro level, the TPU-treated PPy/PDA/SF does not shed PPy after compression (Figure S1). This is because the TPU acts as an adhesive covering for the fibers, making the PPy layer less susceptible to damage by external mechanical action. At the same time, TPU/PPy/PDA/SF is flexible and can be flattened, folded, and curled.

**Figure 4:**
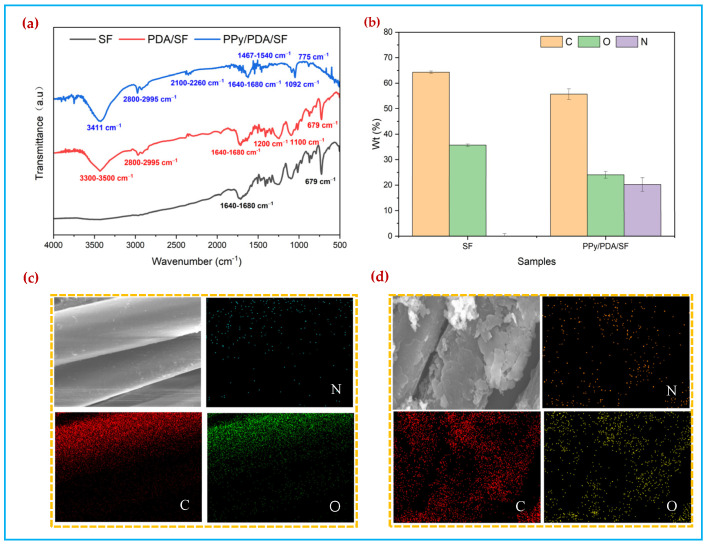
(**a**) FTIR spectra of SF, PDA/SF and PPy/PDA/SF, (**b**) Elemental Quantitative Analysis, (**c**) EDS elemental analysis of SF, (**d**) EDS elemental analysis of PPy/PDA/SF.

Figure 4a illustrates the chemical composition of SF, PDA/SF, and PPy/PDA/SF based on FTIR spectra. The main peaks of SF characterize the C, O, and H molecules as referring to C-C out-of-plane ring vibration (679 cm^−1^) and C=C bonds (1640–1680 cm^−1^) [37,38]. Referring to the spectra of the SF, PDA/SF shows the newly formed N–H bonding (3300–3500 cm^−1^) and its tendency to increase the level of C–OH (1200 cm^−1^) and C–O–C (1100 cm^−1^) bonding depositions and stretching vibration of C-H bonds (2800–2995 cm−1) respectively, indicating successful coating of PDA [39]. The PPy polymerization is recognized as a complex reactive process that includes oxidation, deprotonation, and crosslinking preparations of PPy [40]. Figure 4a shows that the successful deposition of PPy on PDA/SF with different coating types was observed, with similar clear line pattern in-between, and sharp main highlights representing C, N, and H bond structures. This was proved with the peak lines referring to bending vibrations of PPy (775 cm^−1^), N–H plane deformations (1092 cm^−1^), Py ring fundamental vibrations (1467–1540 cm^−1^), C=C bonds (1640–1680 cm^−1^), C=C & C=N bonds (2100–2260 cm^−1^), and N–H bonds (3411 cm^−1^) as highlights [37]. It can be demonstrated that polypyrrole has been polymerized on the fabric.

Furthermore, energy dispersive spectroscopy analysis (Figure 4b) shows that, compared with the uncoated SF, N elements belonging to PPy appear in the coated fabrics. Different from the uniform and smooth distribution of C and O on SF, C, O, N elements are distributed in layers on the surface of the fabric, thereby indicating that the PPy coating has been successfully deposited onto the SF (Figure 4b–d).

### 3.3. Electrical Conductivity

**Figure 5:**
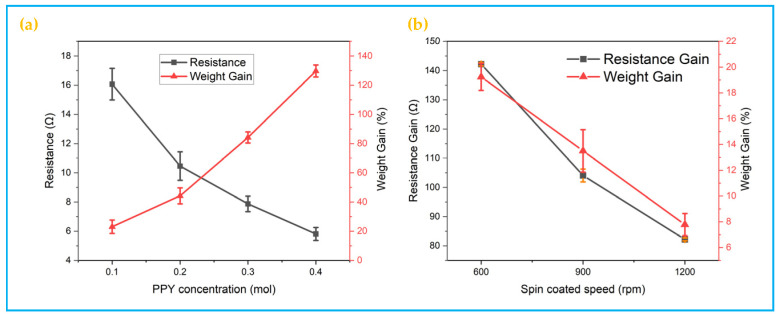
Electrical conductivity; (**a**) Resistance and mass gain of conductive fabrics with different concentrations of pyrrole polymerization; (**b**) Resistance Gain and mass gain of TPU spin-coated at different speeds.

The conductivity of SF/PDA/PPy composite fabric has good electrical conductivity as can be seen from Figure 5a, which because after in-situ polymerization of polypyrrole the spaced fabric surface yarns have been covered by polypyrrole coating with good electrical conductivity, and the polypyrrole coated yarns are in contact with each other to form a good electrical conductivity network. In addition, the conductivity of PPy/PDA/SF increases with the increasing concentration of Py, as shown in Figure 5a. When the concentration of Py increases from 0.1 mol to 0.4 mol, the bulk resistance of PPy/PDA/SF decreases sharply from 16 Ω to 6 Ω (as shown in Figure 5a), which correlates with the attachment of polypyrrole on the fiber, exactly corresponding to the weight of PPy/PDA/SF which increases inversely.

Although PPy/PDA/SF has excellent flexibility and electrical conductivity, the weaker mechanical strength affects its application. Regarding TPU as a block linear polymer, there are many physical crosslinks between polyurethane molecular chains consisting of hydrogen bonds, which strengthen its morphology and thus give many excellent properties, such as high modulus, high strength, excellent friction, chemical resistance, hydrolysis resistance, high and low temperature resistance, and mold resistance [41], which are expected to improve the stability of PPy/PDA/SF. Figure 5b shows the resistance and weight gain quality of TPU/PPy/PDA/SF with different spin-coating speeds. It can be seen that the combination of TPU with the polypyrrole layer on the surface of PPy/PDA/SF fabric by dipping spin-coating significantly improves the resistance of PPy/PDA/SF. The resistance of the TPU/SF/PPy composite fabric prepared by impregnating and spin-coating TPU increased to some extent, mainly because the conductive network was partially isolated by the TPU coating on the surface of the polypyrrole coating. With the increase of spin-coating speed, the resistance increases and weight gain of TPU/PPy/PDA/SF both tend to decrease, mainly because the higher the spin-coating speed, the less TPU remains on the surface of the fabric. Therefore, the resistance increases and weight gain tends to decrease.

### 3.4. Pressure Sensing Characteristics of the PPy/PDA/SF Composite

The pressure sensors were fabricated by fixing PPy/PDA/SF and TPU/PPy/PDA/SF between copper sheet electrodes. To investigate the effect of fabric tissue on the piezoresistive performance, pressure sensors with different pyrrole concentrations and different spin-coating speeds were prepared and tested using a flexible sensor performance testing system. As a basic parameter of the pressure sensor, the sensitivity (S) of the pressure sensor was calculated according to the following Equation [4]

(1) $$S=\delta \left(∆R/R_{0}\right)/\delta P$$

(2) $$∆R=R-R_{0}$$

where R and $R_{0}$ are the real-time resistance and initial resistance of the sample, respectively, and ∆P denotes the applied pressure.

**Figure 6:**
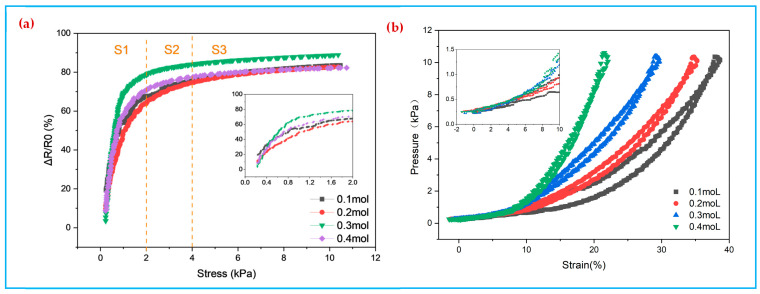
(**a**) Relative rate of change of resistance of PPy/PDA/SF with pyrrole concentrations (0.1–0.4 mol), (**b**) Stress-strain relationship for PPy/PDA/SF with pyrrole concentrations (0.1–0.4 mol).

Figure 6a shows the curves of the relative rate of change of resistance of the polypyrrole dopamine coated fabric with the pressure. The relative rate of change of resistance increases monotonically with the increase of pressure. With the increase of pressure, the relative resistance rate of change tends to rise, and when the pressure reaches 3 kPa, the rising curve of the relative resistance rate of change gradually becomes slower. The highest sensitivity of the pressure sensor was 56.40 kPa^−1^, 57.97 kPa^−1^, 77.04 kPa^−1^, and 68.16 kPa^−1^ when the concentration of Py was 0.1–0.4 mol. The 0.3 mol PPy/PDA/SF composite fabric showed the highest sensitivity of 77.04 kPa^−1^, which is higher than the most reported results in literature shown in Table 1. The sensing range can be divided into three regions, which are 0.155–2 kPa, 2–4 kPa and 4–10 kPa, the corresponding sensitivities are 77.04 kPa^−1^, 7.52 kPa^−1^, 2.07 kPa^−1^, respectively. Figure 6b shows the stress-strain curves of different pyrrole samples at a pressure of 0–10 kPa. The PPy/PDA/SF composite fabrics were compressed between 15% and 40%. When the compression is the same, the higher the stress of compressing the same displacement, the stiffer the fabric is. As the concentration of Py increases, the hardness of PPy/PDA/SF composite fabric tends to rise, while the hysteresis tends to fall. The minimum hysteresis is 4.6% at a Py concentration of 0.4 mol, 4.7% at a pyrrole concentration of 0.3 mol, and 11.8% at a concentration of 0.1 mol. The detailed calculation data are shown in Supplementary Material Table S2. Therefore, considering the results of sensitivity, softness, and hysteresis and combining with SEM images of electron microscopy, a Py concentration of 0.3 mol was selected as the best process parameter.

Table 1 shows the comparison of the main performances of piezoresistive sensors reported by recent literature. It proved the feasibility of fabricating highly sensitive piezoresistive sensors by coating PPy with highly breathable spacer fabrics.

**Figure 7:**
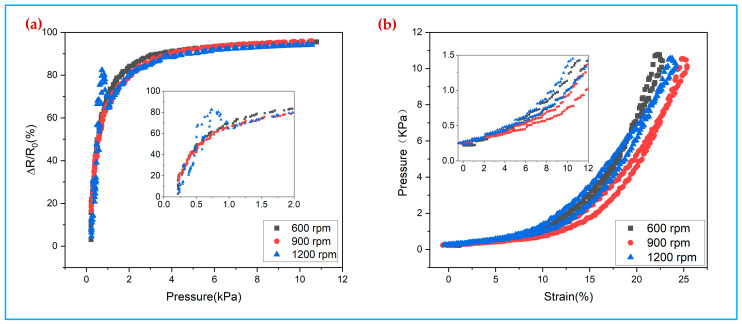
(**a**) Relative rate of change in resistance of spin-coated TPU conductive fabric at different speeds, (**b**) deformation-load relationship of spin-coated TPU conductive fabric at different speeds.

Figure 7a shows the relative rate of change curves of the resistance of the TPU/PPy/PDA/SF sensor at different spin-coating speeds. The TPU/PPy/PDA/SF flexible piezoresistive sensor has a wide operating range 0–10 kPa and is more sensitive at low pressures.

Comparing the data of different spin-coating speeds, it can be found that the stability of the sensors prepared at 900 rpm is best, followed by 600 rpm, while the stability of the 1200 rpm sensor is poor, and the volatility of the data during the measurement process is large. This is because at the spin-coating speed of 900 rpm, TPU is evenly coated to the three-dimensional spaced fabric fiber surface, while at the spin-coating speed of 600 r/min, TPU appears as a large area of agglomeration leading to uneven coating on the fiber surface. When the spin-coating speed reaches 1200 rpm, the TPU on the fabric surface is thrown away from the fabric surface by centrifugal force, which cannot achieve good coating effect, and at the same time, the spin-coating speed is too large to lead to the peeling of the pyrrole coating. Therefore, the sensing performance of TPU/PPy/PDA/SF composite fabric is better when the spin-coating speed is 900 rpm. Figure 7b shows the stress-strain curves of the composite fabrics prepared by different spin-coating speeds, from which it can be seen that the compression of the TPU/PPy/PDA/SF composite fabric pressure sensor is (20–25)% when the pressure is 0–10 kPa. The composites fabric prepared by spin-coating at 900 rpm has the best softness, while different spin-coating speeds (600, 900, and 1200 rpm), the hysteresis is 2.1%, 3.2%, and 5.3%, respectively, as shown in Table S3 of the Supplementary Material. Hereby on comprehensive consideration, 900 rpm of spin-coating is the optimal process parameter.

**Figure 8:**
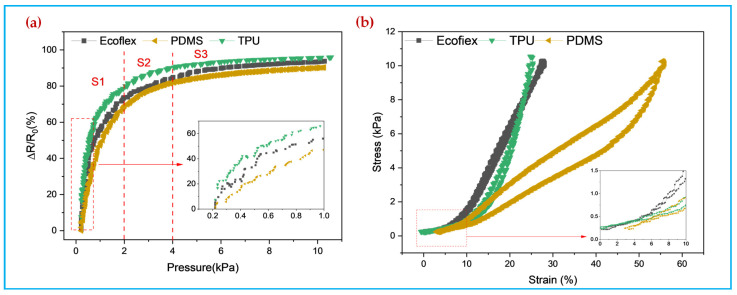
(**a**) Relative rate of change of resistance of TPU900/PPy/PDA/SF, PDMS900/PPy/PDA/SF and Ecoflex900/PPy/PDA/SF (**b**) Stress-stain curve of PPy/PDA/SF at 900 rpm for different rubber polymers.

Figure 8a shows the relative resistance change rate of the sensors when different polymers are spin-coated at 900 rpm, from which it can be seen that the TPU900/PPy/PDA/SF pressure sensor prepared after spin-coating TPU has the highest sensitivity (97.28 kPa^−1^), while the PDMS900/PPy/PDA/SF and Ecoflex900/PPy/PDA/SF pressure sensors have sensitivities of 72.52 kPa^−1^ and 84.30 kPa^−1^, respectively. Figure 8b shows the stress-strain curves of TPU900/PPy/PDA/SF, PDMS900/PPy/PDA/SF, and Ecoflex900/PPy/PDA/SF composite fabrics. The PDMS900/PPy/PDA/SF fabric compresses by more than 50% at a pressure of 0–10 kPa, indicating that this sensor is the softest, but with the greatest hysteresis, due to the fact that PDMS itself has some viscosity and cannot recover quickly to its initial state. The TPU900/PPy/PDA/SF and Ecoflex900/PPy/PDA/SF compresses are less compressed and have a much smaller hysteresis of 3.2% and 2.1% compared to the 27.8% of the PDMS900/PPy/PDA/SF. The rate of change of resistance of the sensor in the range of 0–10 kPa, the change of resistivity can be clearly divided into three segments, so the pressure range of 0–10 kPa is divided into three different regions corresponding to different slopes, i.e., sensitivity. As in Table S3, the sensitivity at a pressure of 0–2 kPa is as high as 97.28 kPa^−1^, the sensitivity in the pressure range of 2–4 kPa can reach10.25 kPa^−1^, and the sensitivity at a pressure of 4–10 kPa reaches 1.55 kPa^−1^, indicating that the PPy dopamine-coated fabric spin-coated with TPU can effectively improve the sensitivity of sensor.

**Figure 9:**
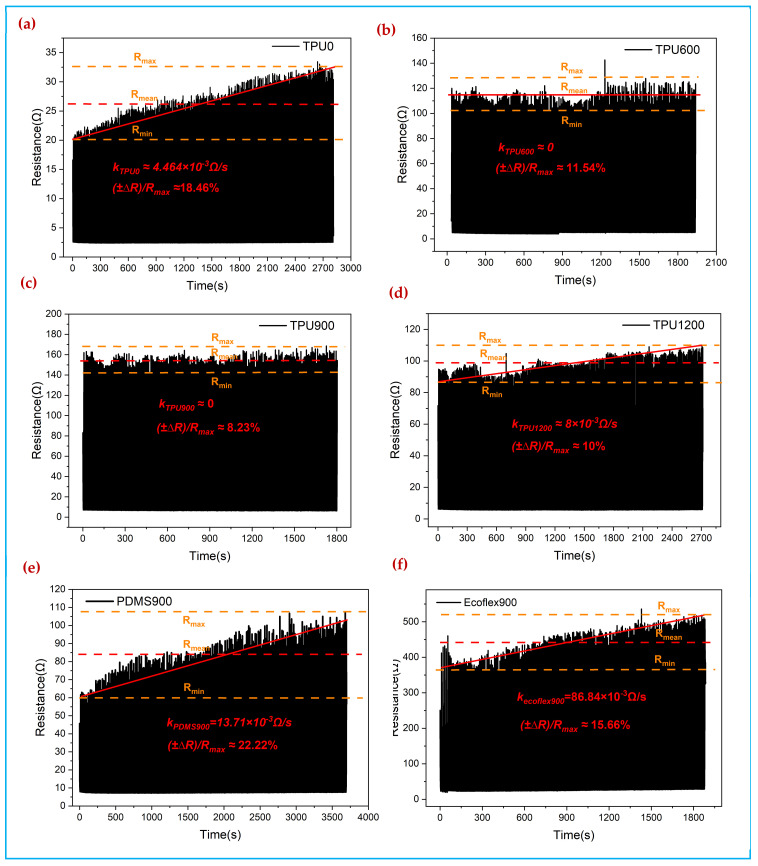
Repeat stability tests for 0.3 mol pyrrole concentration coated with different polymers and different spin-coating speeds; (**a**) without polymers, (**b**) TPU with 600 rpm, (**c**) TPU with 1200 rpm, (**d**) TPU with 900 rpm, (**e**) PDMS with 900 rpm, (**f**) Ecoflex with 900 rpm.

Figure 9 shows the relative rate of change curve of resistance of PPy/PDA/SF fabric impregnated with different polymers and spin-coated with a copper foil electrode to form a pressure sensor over 500 load-unload cycles. The pressure applied was 0–6 kPa and the compression rate was 0.1 mm/s. In this paper, the slope of the curve fluctuation *k* and fluctuation rate $\frac{\pm ∆R}{R_{m}}$ are used to indicate the cycle stability of the composite fabric. The smaller the slope, the better the cyclic stability of the composite fabric; the smaller the fluctuation rate of the curve when the slope is close to 0 the better the stability of the sensor. From Figure 9a–d, the repeat stability of SF/PDA/PPy fabric is poor, and the resistance is gradually becoming larger during the cyclic compression. The slope of the cyclic stability curve decreases after spin-coating with 4% wt. TPU, the slope is close to 0 when the spin-coating speed is 600 rpm and 900 rpm, and the fluctuation of the curve is smaller at 900 rpm (8.23%) than at 600 rpm (11.54%). After spin-coating PDMS and Ecoflex, the cyclic curves of the composite fabrics showed larger slope values, which proved that the coating of PDMS and Ecoflex did not effectively improve the cyclic stability of the composite fabrics. The reason is that TPU can form a thin protective film evenly on the surface of PPy/PDA/SF fabric, while PDMS and Ecoflex, due to their viscous nature, adhere to the three-dimensional structure of the composite fabric after coating, resulting in a viscous appearance of the fabric as a whole, leading to poor cycling stability. Further combined with the electron micrographs, sensitivity, and other parameters, it can be seen that the TPU900/PPy/PDA/SF composite fabric obtained after spin-coating TPU at 900 rpm has the best sensing performance.

### 3.5. Stability and Application

**Figure 10:**
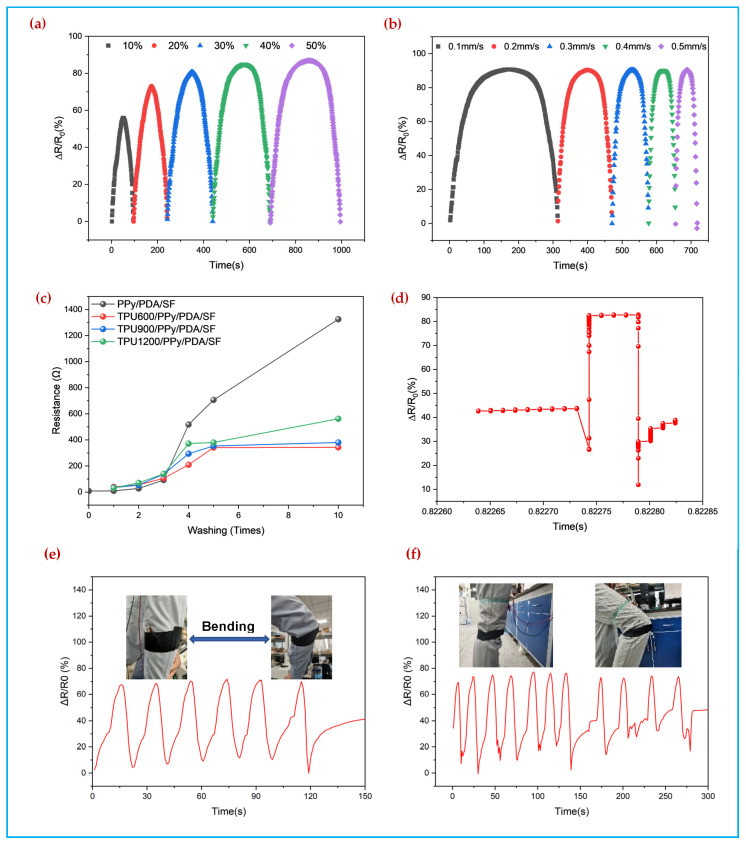
(**a**) Relative rate of change at different compression deformation of TPU coated at 0.3 mol pyrrole concentration 900 rpm, (**b**) Relative rate of change at different compression speeds of TPU coated at 0.3 mol pyrrole concentration 900 rpm (**c**) Resistance change after washing with different spin-coating speeds (**d**) Response time (**e**) leg application of TPU coated at 0.3 mol pyrrole concentration 900 rpm (**f**) arm application of TPU coated at 0.3 mol pyrrole concentration 900 rpm.

Due to the high elastic recovery properties of the spacer fabric, the pressure transducer has a fast and stable response at different mechanical frequencies and amplitudes. As shown in Figure 10a, the pressure transducer has a stable and continuous response to various loads in the compression range of 10%, 20%, 30%, 40%, and 50%. Figure 10b shows the stable response of the pressure transducer at different compression frequencies in the range of 0.1 mm/s, 0.2 mm/s, 0.3 mm/s, 0.4 mm/s, 0.5 mm/s. Figure 10c shows the change in resistance of the fabric after washing. When the water washing reaches the third time, the resistivity of the 0.3 mol PPy/PDA/SF fabric starts to increase gradually. The TPU/PPy/PDA/SF fabrics with spin-coating speeds of 600 rpm and 900 rpm showed less change in resistivity, proving that the polypyrrole coated fabric is severely damaged during the water wash, affecting the electrical conductivity. The PPy/PDA/SF fabric underwent shedding of PPy during the water wash, disrupting the continuity of its conductive structure. TPU is wrapped tightly around the polypyrrole surface like a layer of glue, which plays a certain protective role for the pyrrole layer [40], so the fabric resistance changes more smoothly after spin-coating the TPU. Figure 10d shows the pressure sensor response time of 60 ms at an external pressure of 0–2 kPa, indicating the fast response of the pressure sensor of TPU/PPy/PDA/SF. Figure 10e shows an optical image of the knee attached and the corresponding resistance change signal at different bending angles. The resistance of the pressure sensor decreases when the knee is bent, as the squeezing of the sensor during the bending process exerts pressure on the sensor. In Figure 10f, the fabric pressure sensor is attached to the arm to monitor the pressure difference in muscle movement during bending of the human arm, and the fabric pressure sensor shows a significantly consistent signal over the course of five bends, which can realize the function of human motion monitoring. The sensor has a certain application potential in the field of flexible wearable electronics and can be used to detect pressure changes in real time.

## 4. Conclusions

In this study, a TPU/PPy/PDA/SF composite fabric piezoresistive sensor with high permeability, high sensitivity, and stable sensing performance was successfully prepared using in situ polypyrrole coating and impregnated spin-coated TPU. The mechanical properties of the spacer fabric showed that the SF-X fabric has a larger pressure measurement range (18 kPa) and lower hysteresis repeatability (17.46 ± 8.28) %, which make it more suitable for use as a substrate material for pressure sensors. The best overall performance of the PPy/PDA/SF conductive spacer fabric was achieved at a pyrrole concentration of 0.3 mol and the polymerization time of 60 min, with uniform PPy coating coverage on the fabric surface, low resistance (0.12467 ± 0.00709) Ω, high sensitivity (77.04.65 kPa^−1^), and still good fabric softness and high permeability (3987.9 mm/s, which is about eight times that of cotton fabric). The PPy/PDA/SF conductive spacer fabric was further impregnated and spin-coated with different polymers (TPU, PDMS, and Ecoflex), which showed that TPU effectively improved the cycling stability and mechanical durability of the sensor. The TPU/PPy/PDA/SF pressure sensor prepared by spin-coating at 900 rpm has the best sensing performance, wide working range (0–10 kPa), high sensitivity (97.28 kPa^−1^), fast response (60 ms), good cycling stability (>500 cycles), and real-time monitoring the motion of different body parts (e.g., arms and knees).

## Figures and Tables

**Table 1:** The sensitivity of piezo-resistive sensor with different preparation methods.

Refs: No.	Materials	Method	Linear Range kPa	Highest Sensitivity (kPa^−1^)
[2]	MXene/cotton fabric	dip-coating and sandwiched between PDMS	0–1.30 0–160	5.30 -
[3]	PU@CNT films	Solution modification	0–3 kPa 0–16 kPa	51.53 -
[6]	AgNWs/PU sponge	dip-coating and precompression treatment	-	26.07
[12]	PPy/PDMS	Oxidative polymerization	0–1	19.32
[15]	m-MWNT/PU	Solution modification	0–63	4.282
[29]	MPS	In situ polymerization of polypyrrole	1 kPa 1–10 kPa	10.69 ± 1.53 7.20 ± 2.46
This Study	Space Fabric	In situ polymerization and spin-coating TPU	0–2 kPa 2–4 kPa 4–10 kPa	97.28 10.25 1.55

## Data Availability

Not applicable.

## Supplementary Materials

The following supporting information can be downloaded at: https://www.mdpi.com/article/10.3390/polym14050859/s1, Figure S1: Comparison of coating powder drop on PPy/SF, PPy/PDA/SF and TPU/PPy/PDA/SF after 10 times pressing; Table S1: Performance parameters of different spacer fabrics; Table S2: Table S2 Sensing performance parameters of different concentration sensors; Table S3: Sensing performance parameters of sensors with different spin coating speeds.
