# Supplementary material for: Development of High-Sensitivity Piezoresistive Sensors Based on Highly Breathable Spacer Fabric with TPU/PPy/PDA Coating
Source: Polymers (Basel). 2022 Feb 22;14(5):859. doi: 10.3390/polym14050859 (PMC8912863; doi:10.3390/polym14050859)
Supplement: Supplementary file 1 [file polymers-14-00859-s001.zip › Supplementary material.docx-final.pdf]

Supplementary Material

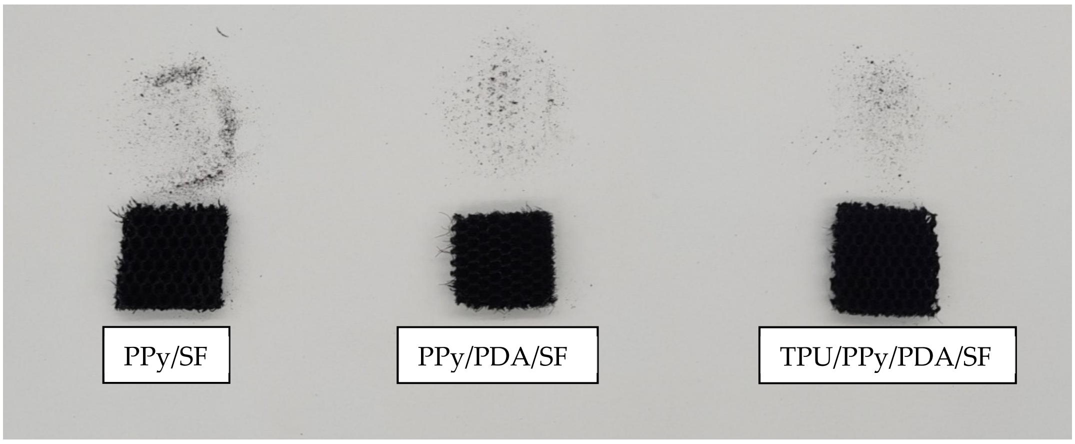

Figure S1. Comparison of coating powder drop on PPy/SF, PPy/PDA/SF and TPU/PPy/PDA/SF after 10 times pressing.

Table S1. Performance parameters of different spacer fabrics.

	Hysteresis		Zero drift		Pressure（kPa）	
	SF-L	SF-X	SF-L	SF-X	SF-L	SF-X
10%	7.05	3.04	1.58	2.85	0.6375	2.480
20%	24.03	17.98	2.36	0.7	3.576	6.550
30%	29.09	22.36	6.01	0.98	5.674	8.550
40%	27.94	22.8	2.26	1.37	6.595	13.862
50%	30.95	21.1	2.95	1.3	7.865	17.950

Table S2. Sensing performance parameters of different concentration sensors.

PPy concentration (mol)	Hysteresis (%)	Sensitivity （KPa^{-1}）	Pressure （kPa）
0.1	11.8	56.40	10
0.2	7.14	57.96	10
0.3	4.7	77.04	10
0.4	4.6	68.16	10

Relative rate of change of resistance for conductive spacer fabrics with pyrrole concentrations of 0.1 mol, 0.2 mol, 0.3 mol and 0.4 mol

$$\begin{cases} y_1 = -18.53315x^2 + 65.11227x + 8.49913, & R^2 = 0.98151 \\ y_2 = -16.93159x^2 + 65.65782x - 0.82505, & R^2 = 0.99569 \\ y_3 = -25.92944x^2 + 85.079288x + 0.98044, & R^2 = 0.97758 \\ y_4 = -20.25242x^2 + 77.88361x + 0.51959, & R^2 = 0.98254 \end{cases} \tag{1-1}$$

According to formula (1-1), we can get：

$$\begin{cases} y_1{}' = -37.0663x + 65.11227 \\ y_2{}' = -33.86318x + 65.65782 \\ y_3{}' = -51.85888x + 85.079288 \\ y_4' = -40.50484x + 77.88361 \end{cases} \tag{1-2}$$

Table S3. Sensing performance parameters of sensors with different spin coating speeds.

Samples	Hysteresis %	Sensitivity (kPa^{-1})	Pressure (kPa)	Zero drift %
TPU600	2.1	94.71	10	16.8
TPU900	3.2	97.28	10	9.27
TPU1200	5.3	90.32	10	21.1
PDMS900	27.8	72.52	10	9.27
Ecoflex900	2.1	84.30	10	16.8

Relative rate of change of resistance for PPy/PDA/SF coated with TPU, PDMS and Ecoflex at different spinning speed.

$$\begin{cases} y_1 = -35.54147x^2 + 110.16575x - 2.88609, \quad R^2 = 0.96279 \\ y_2 = -36.64471x^2 + 113.95275x - 13.75384, \quad R^2 = 0.97253 \\ y_3 = -32.46053x^2 + 105.74172x - 8.92804\,, \quad R^2 = 0.94322 \\ y_4 = -21.26671x^2 + 81.8774x - 13.11527\,, \quad R^2 = 0.99513 \\ y_5 = -29.67111x^2 + 97.20986x - 9.15125\,, \quad R^2 = 0.96072 \end{cases} \tag{2-1}$$

According to formula (2-1), we can get:

$$\begin{cases} y_1' = -71.08294x + 110.16575 \\ y_2' = -73.28942x + 113.95275 \\ y_3' = -64.92106x + 105.74172 \\ y_4' = -42.53342x + 81.8774 \\ y_5' = -59.34222x + 97.20986 \end{cases} \tag{2-2}$$
